# TNFα Induces Müller Glia to Transition From Non-proliferative Gliosis to a Regenerative Response in Mutant Zebrafish Presenting Chronic Photoreceptor Degeneration

**DOI:** 10.3389/fcell.2019.00296

**Published:** 2019-11-26

**Authors:** Maria Iribarne, David R. Hyde, Ichiro Masai

**Affiliations:** ^1^Okinawa Institute of Science and Technology Graduate University, Okinawa, Japan; ^2^Department of Biological Sciences, University of Notre Dame, Notre Dame, IN, United States; ^3^Center for Stem Cells and Regenerative Medicine, University of Notre Dame, Notre Dame, IN, United States; ^4^Center for Zebrafish Research, University of Notre Dame, Notre Dame, IN, United States

**Keywords:** photoreceptor degeneration, regeneration, Müller glia, rod precursors, Aipl1, genetic mutant, zebrafish

## Abstract

Unlike mammals, zebrafish have the capacity to regenerate neurons in response to damage. Most zebrafish retinal injury models employ acute damage, which is unlike the chronic, gradual damage that occurs in human retinal diseases. Here, we studied the regenerative response in the zebrafish *aipl1b* mutant, *gold rush* (*gosh*). In *gosh* mutants, both cones and rods degenerate by 3 weeks post-fertilization (wpf). Müller glia do not exhibit a regenerative response by 3 wpf; however, they do present non-proliferative gliosis. Only at 5 wpf, is proliferation of Müller cells and rod precursor cells activated. Rods start to recover at 5 wpf and by 12 wpf they reach a level of recovery comparable to wild type, but cones remain absent in the adult stage. TNFα was detected in degenerating cones at 5–7 wpf and in Müller glia at 7 wpf in *gosh* mutants. At 5 wpf, proliferating Müller glia express Sox2, followed by Pax6 expression in neuronal progenitor cells (NPCs), confirming that the neuronal regeneration program is activated in *gosh* mutants after 5 wpf. Although acute light-induced damage did not activate proliferation of Müller glia, TNFα injection caused Müller glia to commence a proliferative response at 3 wpf in *gosh* mutants. These results suggest that Müller glia transition from non-proliferative gliosis to a regenerative state in *gosh* mutants, and that ectopic introduction of TNFα promotes this Müller cell transition even at 3 wpf. Thus, zebrafish *gosh* mutants provide a useful model to investigate mechanisms underlying retinal regeneration in a chronic photoreceptor degeneration model.

## Introduction

Photoreceptor cell death is associated with human genetic diseases of the eye, such as retinitis pigmentosa, Leber congenital amaurosis, and macular degeneration (Sahaboglu et al., 2013; Iribarne and Masai, 2017). The loss of photoreceptors leads to irreversible blindness because regeneration of retinal neurons is extremely limited. Unlike mammals, however, zebrafish exhibit strong regenerative capacity, making them the most popular animal model to study tissue/organ regeneration (Campbell and Hyde, 2017). This retinal regeneration process is dependent on activity of Müller glia (Yurco and Cameron, 2005; Fausett and Goldman, 2006; Bernardos et al., 2007; Fimbel et al., 2007). When neurons are lost in the zebrafish retina, Müller glia are reprogramed to a retinal progenitor cell-like state, whereupon they re-enter the cell cycle and produce neuronal progenitor cells (NPCs) (Iribarne, 2019). These NPCs continue to proliferate and to differentiate into all retinal cell types.

In addition, zebrafish have a unique ability to undergo persistent neurogenesis throughout life. Two mechanisms are responsible for this continuous neurogenesis. Most retinal neurons are produced by a stem cell population that resides in the ciliary marginal zone (CMZ) at the retinal periphery (Johns, 1977). A second population of stem cells, named rod precursors, generates rod photoreceptors in the central retina (Johns, 1977). These rod precursors arise from Müller glia, which slowly divide in the inner nuclear layer (INL). These progenitors then migrate to the outer nuclear layer (ONL) where photoreceptors are located and become rod precursors (Johns, 1982; Otteson and Hitchcock, 2003). Damage to the retina induces a much more pronounced proliferative response by Müller glia to replenish lost neurons. Additionally, if photoreceptors are damaged, rod precursors are also involved in the regenerative response. Investigations into behaviors of different progenitor cell populations following retinal injury should illuminate mechanisms underlying regenerative responses.

Most previous regeneration studies have employed acute damage that injures the adult retina using strong light exposure (Vihtelic and Hyde, 2000; Bernardos et al., 2007), retinal puncture (Fausett and Goldman, 2006), chemical ablation (Fimbel et al., 2007), or ectopic expression of a toxic transgene, such as nitroreductase (Montgomery et al., 2010; Hagerman et al., 2016). Such acute damage results in rapid loss of retinal cells that resembles traumatic injury in human patients. While each of these damage models eliminates different retinal cell types, they all elicit a similar response in Müller glia to regenerate lost neuronal cell types. Several secretion molecules that participate in Müller glia dedifferentiation and proliferation have been identified, including TNFα (Nelson et al., 2013), HB-EGF (Wan et al., 2012), TFGβ (Lenkowski et al., 2013), insulin, and Fgf2 (Wan et al., 2014). In addition, Müller glia activate transcription factors that promote retinal cell proliferation and differentiation, such as Ascl1a (Ramachandran et al., 2010), Stat3 (Kassen et al., 2007; Nelson et al., 2012), Pax6 (Raymond et al., 2006; Thummel et al., 2010), and Lin-28 (Ramachandran et al., 2010). However, for modeling human genetic diseases, which usually exhibit a chronic time course that may require years or decades to cause a noticeable loss of neuronal cells and concomitant vision loss, different zebrafish models need to be generated to study the effects of regeneration on chronic neuronal cell loss.

To date, few studies have employed zebrafish photoreceptor genetic mutants to characterize neuronal regeneration (Morris et al., 2008; Nishiwaki et al., 2008; Iribarne et al., 2017). Morris et al. (2008) used zebrafish mutant strains that exhibit very rapid loss of photoreceptors, and observed that regeneration started as early as 1 week post-fertilization (wpf). A non-sense mutation of zebrafish *cGMP phosphodiesterase 6c* (*pde6c*) mutants, *pde6c^*w*59^*, showed acute photoreceptor degeneration and stimulated Müller glia proliferation, whereas a zebrafish transgenic line with acute rod degeneration, MOPS-mCFP, mainly stimulated rod precursor cell proliferation (Morris et al., 2008). *gold rush* (*gosh*) mutants exhibit no visual behavior when evaluated by optokinetic response (OKR), because they harbor a mutation of the cone-specific *arylhydrocarbon receptor interacting protein like 1* (*aipl1*) called *aipl1b* (Iribarne and Masai, 2018). In contrast to the *pde6c^*w*59^* mutant, *gosh* mutant underwent slower progressive photoreceptor cell degeneration that did not stimulate either Müller glia or rod precursor cell proliferation at an early larval stage (1 wpf) (Iribarne et al., 2017). How these and other chronic degeneration mutations cause cell death and affect Müller glia reprograming and proliferation is critical to understand the potential of Müller glia to respond to chronic retinal damage in humans.

This study examined the retinal regeneration process in zebrafish chronic photoreceptor degeneration mutants, *gosh*. We previously described striking behavior in the number of rod photoreceptors in *gosh* mutants (Iribarne et al., 2017). At 4 wpf, the photoreceptor layer in *gosh* mutants is thinner than in wild-type siblings, indicating that both rod and cone photoreceptors undergo degeneration. In contrast, the rod photoreceptor layer in *gosh* mutant adult retinas has relatively normal morphology, but lacks nearly all cones, suggesting that rod photoreceptors are recovered by regeneration. Here, we document regenerative responses of Müller glia and rod precursors in *gosh* mutants.

## Materials and Methods

### Ethics Statement

All zebrafish experiments performed at the Okinawa Institute of Science and Technology Graduate School (OIST) were carried out in accordance with the OIST Animal Care and Use Program, which is based on the Guide for the Care and Use of Laboratory Animals by the National Research Council of the National Academies and which is accredited by the Association for Assessment and Accreditation of Laboratory Animal Care (AAALAC International). All experimental protocols were approved by the OIST Institutional Animal Care and Use Committee (Approval ID: 2014-83∼86). All experiments performed at the University of Notre Dame were approved by the animal use committee at the University of Notre Dame and comply with the ARVO statement for the use of animals in vision research.

### Fish

Zebrafish (*Danio rerio*) were maintained according to standard procedures (Westerfield, 1993). Okinawa wild type (*oki*) was used as a wild-type strain. The *gold rush* mutant was originally isolated in a screen of zebrafish visual mutants using a chemical mutagen, N-ethyl-N-nitrosourea (ENU) (Muto et al., 2005). A zebrafish transgenic line Tg(*gnat2:GFP*)^oki061^ was established to monitor cone photoreceptor integrity. We utilized the transgenic line Tg(*gfap:GFP*)^nt11^ to visualize zebrafish Müller glia (Kassen et al., 2007), and Tg(*zop:nfsB-eGFP*)^nt19^ to visualize rod photoreceptors (Montgomery et al., 2010). All experiments were all least carried out three times.

### Optokinetic Response (OKR)

Optokinetic response was performed to identify visual mutants at 5–7 days post-fertilization (dpf) following a published method (Iribarne et al., 2017). In a petri dish containing methylcellulose, 10 wells were filled with water from the aquarium in which the fish were raised, so as to minimize stress to the fish. Each well accommodated one larva which was partially immobilized to allow examination under a stereoscopic microscope. To evaluate visual acuity, a drum with black and white vertical stripes (at 18° separation) was placed around the petri dish, and spun at 10–20 rpm. Larval eye movement was observed under the stereoscopic microscope to identify cone blind fish.

### Light Damage Protocol

To induce retinal damage, at 3 wpf, five larvae were placed in a clear glass beaker with 60 mL of system water, illuminated by four fluorescent bulbs (15,000–20,000 lux) for 18 h. This light treatment started at 6 pm and finished at 12 am. Fish eyes were enucleated and immediately fixed in 4% PFA overnight. Afterward, they were processed for immunohistochemistry.

### TNFα Production and Intraocular Injection

TNFα was synthesized according to a published method (Conner et al., 2014). Briefly, the pQE30 plasmid containing recombinant zebrafish TNFα cDNA was transfected into M15 cells (QIAGEN), and recombinant TNFα protein was purified using a QIAExpressionist kit (QIAGEN). Purified TNFα was diluted to a working concentration of 0.5 mg/mL with sterile PBS. TNFα solution (0.5–1 nL) was injected intravitreally into the eyes of 3 wpf *gosh* mutants and wild-type siblings using a FemtoJet express microinjector (Eppendorf). Since 3-wpf larval fish show variable body size, we selected average-sized fish from each genotype group for injection. Two rounds of injection were applied intravitreally every 12 h, and fish were sacrificed 12 h later (24 h after the first injection). Samples were immediately fixed in 4% PFA and processed for immunohistochemistry.

### TUNEL

Cryosections from sibling and *gosh* mutant retinas were used to evaluate cell death. TUNEL was performed using an *In Situ* Cell Death Detection Kit (Roche) and counterstained with TO-PRO-3. The protocol was performed following the manufacturer’s instructions.

### EdU Labeling

A total of 3 wpf old fish were immerse in 1 mM EdU (5-ethynyl-20-deoxyuridine) bath during 2 h pulse and then washed out to labeling cell proliferation. Fish were sacrificed 3 days later, fix in 4% PFA and process for EdU detection. EdU detection was performed using Click-iT EdU Alexa Fluor 594 Imaging Kit (Invitrogen) and counterstained with DAPI. The protocol was performed following the manufacturer’s instructions.

### Histology

Immunolabeling of cryosections and paraffin sections was performed as described previously. Paraffin sections were pretreated at 120°C for 20 min in 10 mM citrate buffer pH 6.0. zpr1 antibody (ZIRC, Eugene, Oregon; 1:100), anti-zebrafish rhodopsin (1:5000), proliferating cellular nuclear antigen (PCNA) (clone PC10, Sigma P8825; 1:200), zrf1 antibody (ZIRC, Eugene, Oregon; 1:100), tumor necrosis factor α (TNFα) (AnaSpec; 1:50), glutamine synthetase (GS) (MAB302, clone GS-6, Millipore; 1:100), Sox2 (AF2018, R&D Systems; 1:100), and Pax6 (PRB-278p-100, BioLegend, 1:500) were used. GFP antibody was used to amplify the signal or to detect GFP after antigen retrieval (A11122, Life Technology, 1:200). Nuclear staining was performed using 1 nM TO-PRO-3 (Molecular Probes) or 5 μg/mL DAPI (Invitrogen). Images were scanned using a confocal laser scanning microscope (Carl Zeiss LSM710, and Nikon A1r).

### Quantitative Real-Time PCR

RNA was prepared from 3-, 5-, and 7-wpf sibling and *gosh* mutant fish. 8–10 fish heads (3w) or eyes (5 and 7 wpf) were dissected and pooled. RNA was extracted using TRIzol reagent (Life Technologies). Total cDNA was synthetized from 1 μg of RNA using qScript cDNA SuperMix (Quanta Biosciences, Gaithersburg, MD, United States). Reactions were assembled using PerfeCta SYBR Green SuperMix (ROX; Quanta Biosciences). Primers used in this study were as follows:

18 S (F: 5′ AATTGACGGAAGGGCACCAC, R: 5′ CTAAGA ACGGCCATGCACCA)

TNFα (F: 5′ AGGCAATTTCACTTCCAAG, R: 5′ AGGTCT TTGATTCAGAGTTGTATCC) Gfap (F: 5′ GCAGACAG GTGGATGGACTCA, R: 5′ GGCCAAGTTGTCTCTCTCGATC). Data were acquired using the StepOnePlus Real-Time PCR system (Applied Biosystems, Foster City, CA, United States). Analysis was performed using the Livak 2^–^^Δ^^Δ^^*C*(*t*)^ method.

### Counting Cells

Cells were counted separately in the ONL and the INL from the whole region of sectioned retina, using 2 or 3 retinal sections of the same eye. Cone and rod photoreceptors were counted across a 100-μm horizontal segment in the central retina. Counts were then averaged and SEM was calculated. A minimum of 4 fish were used for the counts for each experiment, with only one eye used per fish. The statistical significance of differences between control and experimental groups was determined for all experiments using either a two-tailed, unpaired Student’s *t*-test to compare two data points or ANOVA with Bonferroni’s *post hoc* test to compare more than 3 points.

## Results

### Cone and Rod Photoreceptors Degenerate at 3 wpf in *gosh* Mutants

To monitor cone photoreceptor integrity, we generated a transgenic line Tg(*gnat2:GFP*), which expresses GFP from the cone-specific promoter, *gnat2* (Kennedy et al., 2007). At 3 wpf, we examined integrity of cones and rods in *gosh* mutant retinas using Tg(*gnat2:GFP*) and anti-rhodopsin antibody (Supplementary Figure S1). At 3 wpf, the ONL was markedly thinner in the central retinas of *gosh* mutants relative to wild-type retinas. *gnat2:GFP* expression level and shape appeared abnormal, suggesting cone degeneration. Rhodopsin expression was also reduced in the central retinas of *gosh* mutants. In the CMZ of wild-type retinas, retinal stem cells generated all retinal cell-types during embryonic stages. In *gosh* mutant CMZs, cone and rod photoreceptors were present and showed moderately normal columnar shapes, indicating that retinal stem cells continue to generate new cones, which degenerate at later stages. Similarly, the transgenic line Tg(*zop:nfsB-eGFP*)^nt19^ showed abnormal rod cell shape in *gosh* mutants (Supplementary Figure S2). These 3-wpf phenotypes are consistent with our previous results that cone photoreceptors undergo degeneration at 1 and 4 wpf, although transient degeneration of rod photoreceptors at 3 wpf was not resolved previously (Iribarne et al., 2017).

### Retinal Apoptosis Occurs at 2–3 wpf in *gosh* Mutants

**FIGURE 1:**
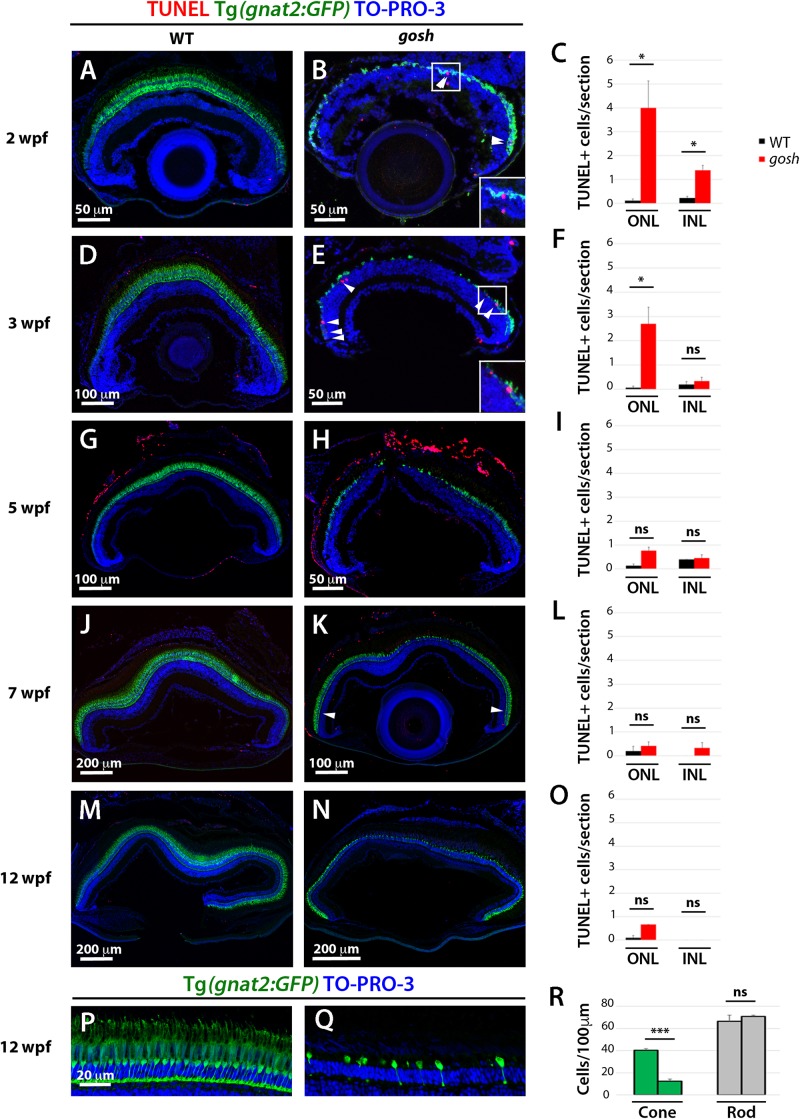
Retinal apoptosis occurs transiently at 2–3 wpf and ceases by 5 wpf in *gosh* mutants. TUNEL was assessed on retinal slides of wild-type sibling **(A,D,G,J,M)** and *gosh* mutant retinas **(B,E,H,K,N)** at 2, 3, 5, 7, and 12 wpf. Apoptotic cells in the ONL are indicated by arrows. The transgenic line Tg(*gnat2:GFP*) is used to label cone photoreceptors. Nuclei are counterstained with TO-PRO-3. Wild-type sibling retinas show very low numbers of TUNEL-labeled cells at all stages **(A,D,G,J,M)**. In *gosh* mutants, numbers of apoptotic cells are higher in the ONL at 2–3 wpf and in the INL at 2 wpf (**B,E**, insets show TUNEL^+^ cells in the ONL). Quantification of TUNEL-positive nuclei in the ONL and INL was performed **(C,F,I,L,O)**. Bars and lines indicate means ± SEM, n: 3*–*7. Black and red bars: wild-type sibling and *gosh* mutants. Central retina of control and *gosh* retinas at 12 wpf show similar rod layer thickness, but cone photoreceptor shows a reduce number of cones (**P–R**, control left bars, *gosh* right bars) (ns, *p* > 0.05; ^∗^*p* < 0.05; and ^∗∗∗^*p* < 0.001). ONL, outer nuclear layer; INL, inner nuclear layer.

We previously reported that TUNEL-positive cells are more abundant in the ONL of *gosh* mutants than in wild-type siblings at 1 wpf (Iribarne et al., 2017). To extend this characterization, we evaluated cell death by TUNEL at later developmental stages in wild-type and *gosh* mutant retinas from 2 to 12 wpf. Cones were visualized using the transgene Tg(*gnat2:GFP*) and nuclei were counterstained with TO-PRO-3. At all stages (2–12 wpf), wild-type retinas showed a continuous cone layer and cones displayed long and thin columnar structure (Figures 1A,D,G,J,M). At 2 and 3 wpf, *gosh* mutant retinas displayed very thin gnat2:GFP-positive cone photoreceptor layers (Figures 1B,E). In these retinas, the cone layer was discontinuous, and severely affected in the central retina. After 5 wpf, cone layers in *gosh* mutants partially recovers in cell number, suggesting that retinal stem cells in the CMZ and Müller cell-mediated regeneration produce new cones in peripheral and central retinas, respectively (Figures 1H,K). However, gnat2:GFP-positive cells form only a single layer in the central retina of *gosh* mutants at 12 wpf (Figures 1N,P,Q), suggesting that continuous cone degeneration occurs among these newly generated cones. This single cone cell layer in *gosh* mutants at 12 wpf was confirmed by our previous electron microscopic analysis (Iribarne et al., 2017).

Rod photoreceptors show abnormal cell shapes and decreased abundance in *gosh* mutants at 3 wpf (Supplementary Figure S2). Indeed, TO-PRO-3+/GFP- rod photoreceptors were less abundant than in wild-type retinas in 2–5 wpf (Figures 1B,E,H and Supplementary Figure S3). Interestingly, TO-PRO-3+/GFP- rod photoreceptor layer thickness started to recover to wild-type thickness (Figures 1H,K,N). We evaluated the number of rods and cones in *gosh* mutant central retinas at 12 wpf, and found that rods show similar nuclear densities in wild-type siblings and *gosh* mutants (wild-type: 66.5 ± 5.69; *gosh*: 71 ± 1.08), whereas cone density was significantly decreased in *gosh* mutants (wild-type: 40.25 ± 1.49; *gosh*: 12.5 ± 1.76) (Figure 1R). These observations suggest that rods degenerate in 2–3 wpf, but that they recover after 5 wpf.

Our previous study revealed that TUNEL-positive cells are significantly more numerous in ONLs of *gosh* mutants than in those of wild-type siblings, but not in the INL (Iribarne et al., 2017). Next, we extended TUNEL to later developmental stages. Wild-type retinas possessed very few TUNEL-positive cells at all ages (2–12 wpf) (Figures 1C,F,I,L,O). In *gosh* mutants, TUNEL-positive cell were observed in the ONL, with a peak at 2 wpf (Figure 1C; ONL WT: 0.11 ± 0.07; *gosh*: 4 ± 1.13), and it remained statistically elevated at 3 wpf (Figure 1F; ONL WT: 0.07 ± 0.07; *gosh*: 2.69 ± 0.68). The number of TUNEL-positive photoreceptors decreased after 5 wpf (Figures 1I,L,O). The INL possessed increased apoptotic nuclei at 2 wpf in *gosh* mutant retinas relative to wild-type retinas (Figure 1C; INL WT: 0.22 ± 0.07; *gosh*: 1.39 ± 0.20), but TUNEL-positive cells were statistically equivalent to wild-type retinas after 3 wpf (Figures 1F,I,L,O). Thus, photoreceptor apoptosis in *gosh* mutants starts at 1 wpf (Iribarne et al., 2017), increases at 2–3 wpf, and ceases after 5 wpf.

### Proliferation of Müller Glia and Rod Precursors Is Activated With a 3-Week Delay After Photoreceptor Degeneration in *gosh* Mutants

**FIGURE 2:**
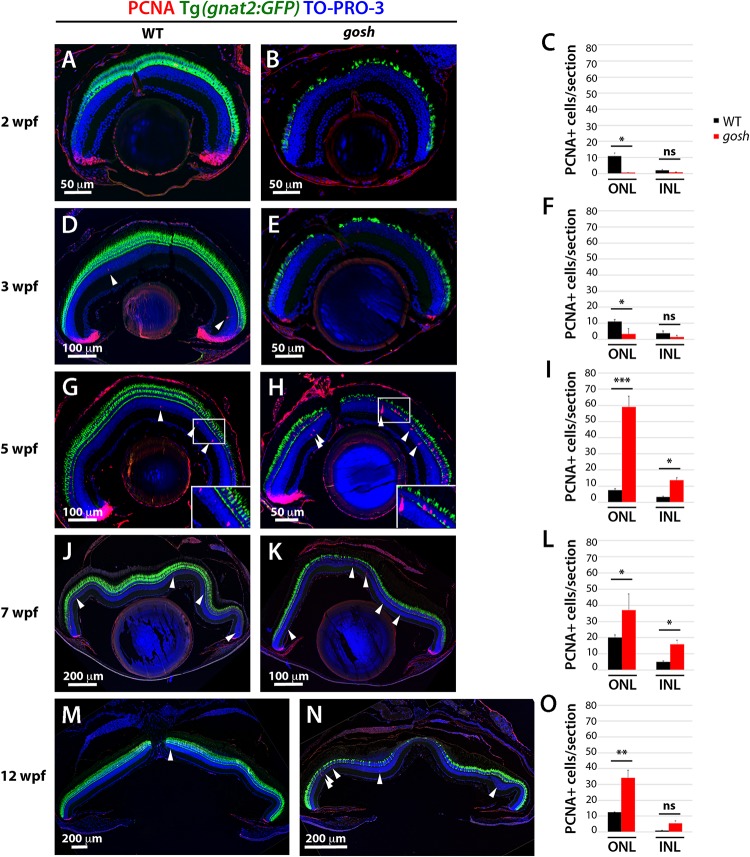
Proliferation of Müller glia, NPCs, and rod precursors starts after 5 wpf in *gosh* mutants. Labeling of wild-type sibling **(A,D,G,J,M)** and *gosh* mutant retinas **(B,E,H,K,N)** at 2, 3, 5, 7, and 12 wpf with anti-PCNA antibody. Cones are visualized with the Tg(*gnat2:GFP*) transgenic line, and nuclei are counterstained with TO-PRO-3. In wild-type retinas, retinal stem and progenitor cells in the CMZ express PCNA, indicating proliferation of retinal progenitor cells. Furthermore, PCNA-positive cells are observed in the ONL and INL, corresponding to rod precursors, and Müller glia/NPCs, respectively, at all stages studied **(A,D,G,J,M)**. In *gosh* mutants, PCNA expression is absent in the CMZ, as well as in the ONL and INL at 2–3 wpf **(B,E)**. At 5 wpf, PCNA-positive ONL and INL cells are drastically increased in *gosh* mutants, suggesting that Müller glia and rod precursor cells start cell proliferation **(H)**. Insets show PCNA-positive cells **(G,H)**. Numbers of PCNA-positive cells in the ONL and INL indicate that proliferation becomes maximal at 5 wpf in *gosh* mutants and maintains higher levels than wild type until 12 wpf **(C,F,I,L,O)**. Bars and lines indicate mean ± SEM, n: 3–9. Black and red bars: wild-type sibling and *gosh* mutant. Arrowhead depict PCNA-positive cells in the INL (ns, *p* > 0.05; ^∗^*p* < 0.05; ^∗∗^*p* < 0.01; and ^∗∗∗^*p* < 0.001).

Because the thickness of the rod photoreceptor layer was restored, we investigated when the regenerative response was activated in *gosh* mutants. We labeled wild-type and *gosh* mutant retinas with anti-PCNA antibody. In wild-type retinas, PCNA expression was primarily restricted to retinal progenitor cells in the CMZ, although it decreased in older fish (Figures 2A,D,G,J,M). In wild-type retina, a small number of PCNA-positive cells were also observed in the ONL and INL, which possibly correspond to Müller glia and rod progenitor cells. These proliferating cells are the source of persistent neurogenesis, where Müller glia divide asymmetrically and infrequently to produce rod progenitor cells, which migrate to the ONL and are committed to differentiate into rod photoreceptors (Lahne et al., 2015). In *gosh* mutants, PCNA expression was drastically reduced in the CMZ and ONL compared with wild type at 2–3 wpf (Figures 2B,C,E,F). We conducted EdU labeling at 3 wpf and confirmed that CMZ retinal progenitors continued to proliferate in *gosh* mutant retinas (Supplementary Figure S4B). To address the relative extent of proliferation in the mutant, we calculated the area of EdU-labeled cells in the CMZ over the total retina area and found that the *gosh* mutants possessed significantly less percentage of EdU-labeled cells in the CMZ (corresponding to a smaller area, Supplementary Figures S4B,D,E) relative to control (Supplementary Figures 4A,C,E). Thus, the CMZ cells in *gosh* mutants continue to proliferate, but at a lower rate, relative to control CMZ.

At 5 wpf, PCNA expression in the retinal CMZ was recovered in *gosh* mutants to a wild-type level. Furthermore, PCNA-positive cells were significantly increased in both ONL and INL of *gosh* mutants, indicating a strong regenerative response (Figures 2H,I; WT ONL: 7.60 ± 0.80, and INL: 3.30 ± 0.50; *gosh* ONL: 59.00 ± 6.56, and INL: 13.81 ± 1.40). PCNA-positive cells were often in clusters that contained proliferating Müller glia and their-derived NPCs. The increase of PCNA-positive INL and ONL cells persisted (Figures 2K,L,N,O). Taken together, these data indicate that photoreceptor cell death at 2–3 wpf was followed by proliferation of Müller glia, NPCs, and rod precursor cells at 5–12 wpf. Hence, proliferation of Müller glia and rod precursors was activated with a 3-week delay following photoreceptor degeneration in *gosh* mutants.

### Müller Glia Display Non-proliferative Gliosis at 3 wpf in *gosh* Mutants

**FIGURE 3:**
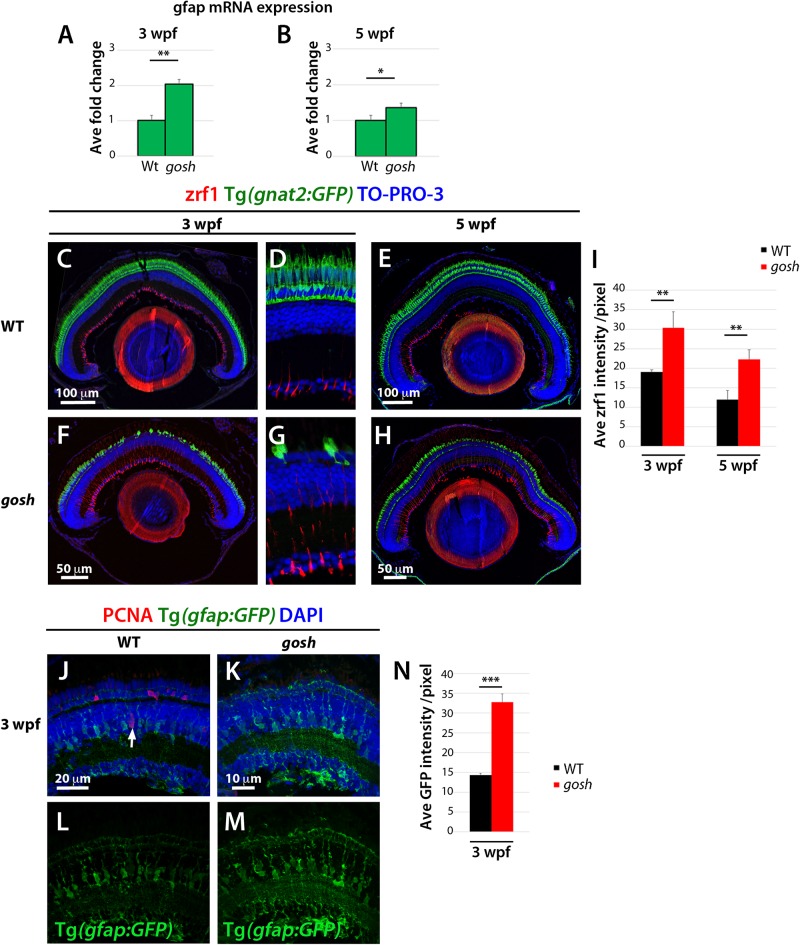
Non-proliferative gliotic response in *gosh* mutants at 3 wpf. **(A,B)** Upregulation of GFAP mRNA expression in *gosh* samples at 3 and 5 wpf compared with control samples. **(C–H)** Paraffin sections labeled with zrf1 antibody, which recognizes GFAP. Cones are visualized with GFP expression of T(*gnat2:GFP*). Nuclei are stained with TO-PRO-3. In wild type, inner radial processes of Müller glia are faintly stained with zrf1 antibody at 3 and 5 wpf **(C–E)**. However, in *gosh* mutants, radial processes of Müller glia are more intensely labeled at 3 and 5 wpf, indicating cell hypertrophy with upregulation of GFAP **(F–H)**. **(I)** Histogram of GFAP-positive area in wild-type and *gosh* mutant retinas at 3 and 5 wpf. GFAP signals are higher in *gosh* mutants than in wild type at both 3 and 5 wpf. **(J–M)** Tg(*gfap:GFP*)^*nt*11^ visualizes Müller glia at 3 wpf. Proliferative Müller glia, NPCs, and rod precursor cells are labeled with PCNA antibody, and nuclei are counterstained with DAPI. In wild-type retinas, GFAP is observed in cell bodies and apico-basal extended processes of Müller glia **(J,L)**. Some Müller glia express PCNA (**J**, arrow). PCNA-positive cells are also observed in the ONL, indicating persistent neurogenesis to produce rod photoreceptors. In contrast, PCNA expression is absent or very low in *gosh* mutants at 3 wpf **(K)**. GFAP is upregulated in Müller cells, which show a greater number of cell processes **(K,M)**. Notice the strong GFP fluorescence in the ONL, where photoreceptors are degenerating. **(N)** Histogram of gfap-positive area in control and *gosh* retinas depicts the increase of fluorescence in the mutant retina.

Persistent reactive gliosis has two forms in mammals, non-proliferative and proliferative. Non-proliferative reactive gliosis is associated with retinal damage and is characterized by persistent upregulation of glial fibrillar acidic protein (GFAP) linked to hypertrophy of Müller glia (Iribarne et al., 2008; Bringmann and Wiedemann, 2012). To assess whether non-proliferative gliosis of Müller glia occurs in *gosh* mutants, we evaluated GFAP mRNA expression by quantitative real-time PCR (qPCR) in samples from 3 and 5 wpf. Baseline levels of GFAP mRNA were observed in wild-type samples, while *gosh* mutant GFAP levels were upregulated at 3 wpf (Figure 3A, WT: 1.01 ± 0.15; *gosh*: 2.04 ± 0.13). *gosh* mutants continue to present upregulated levels of GFAP mRNA relative to control samples at 5 wpf (Figure 3B, WT: 1 ± 0.12; *gosh*: 2.04 ± 0.17). Retinal sections were immunostained against GFAP antibody (zfr1) at 3 and 5 wpf. In wild type, Müller glia showed faint GFAP expression, which only labels their inner radial processes at 3 and 5 wpf (Figures 3C–E,I). In *gosh* mutants, GFAP expression was elevated at both 3 and 5 wpf relative to wild-type control (Figures 3F–I). We also examined morphology of Müller glia at 3 wpf using the Tg(*gfap:GFP*) transgenic line. In wild type, Müller glia show normal morphology in which nuclei are located in the INL with extensions of their apical and basal thin processes (Figures 3J,L). However, in *gosh* mutants, Müller glia show hypertrophic morphology, in which their cellular processes show increased GFP intensity (Figures 3K,M,N). These results suggest that Müller glia undergo non-proliferative reactive gliosis at 3 wpf in *gosh* mutants. Since GFAP expression is enhanced in *gosh* mutant Müller cells at 5 wpf (Figures 3B,H,I), gliosis still persists at 5 wpf, when regenerative cell proliferation begins.

### TNFα Signals From Dying Photoreceptors Induce Proliferation of Müller Glia

**FIGURE 4:**
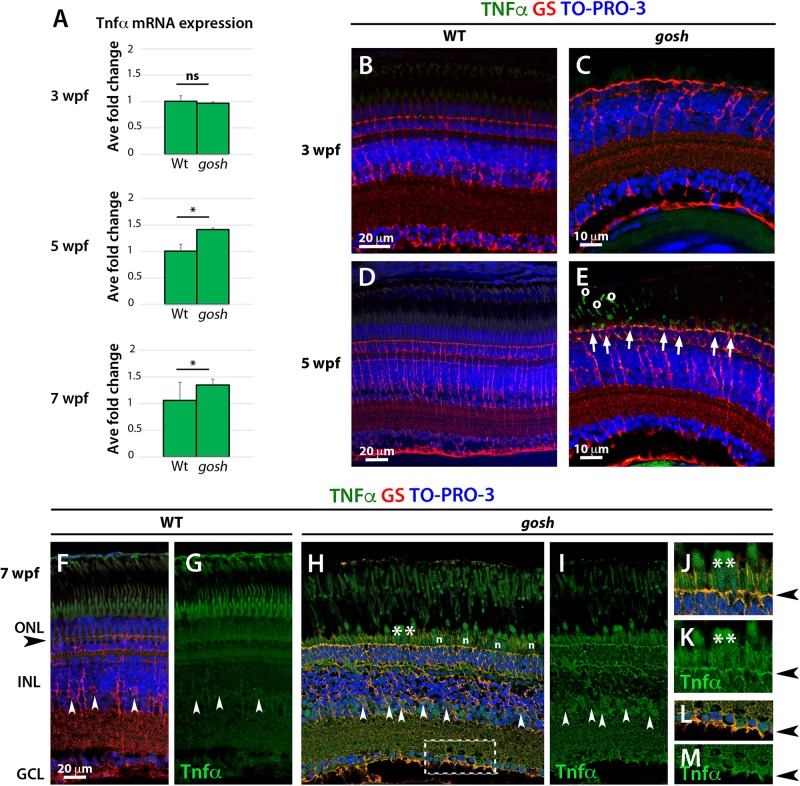
TNFα is detected in cones at 5 wpf and in Müller glia at 7 wpf in *gosh* mutants. **(A)** Quantitative PCR histogram of 3, 5, and 7 wpf of control and *gosh* samples. 3 wpf of control and *gosh* samples displayed similar levels of TNFα mRNA. Upregulation of TNFα mRNA at 5 and 7 wpf in *gosh* samples relative to control samples. **(B–M)** Paraffin sections of wild-type sibling and *gosh* mutant retinas were labeled with antibodies against TNFα and GS at 3, 5, and 7 wpf. Müller glia are visualized as cells with nuclei located in the INL and radial processes that span the apico-basal axis of the neural retina. Wild-type sibling retinas exhibit undetectable levels of TNFα **(B,D)**. *gosh* mutant retinas show no TNFα immunoreactivity at 3 wpf, as in wild-type retinas **(C)**; however, degenerating cones display labeling against TNFα at 5 wpf (**E**, arrows). Circles in **(E)** denote autofluorescence. Nuclei of Müller glia are localized in the INL (white arrowhead) and their basal and apical processes reach the basal region of the retinal ganglion cell layer and the outer limiting membrane (black arrowhead) in the ONL in 7-wpf wild-type retinas **(F,G)**. TNFα immunoreactivity is absent, although background signals are observed in the cone outer segment. However, in *gosh* mutant retina, strong signals are detected in dying cones (**H**, asterisks) and weaker signal in Müller glia (white arrowhead) and their cell process **(H,I)**. **(J,K)** indicate high magnification images of the ONL shown in (**H**, asteriks). **(L,M)** indicate high magnification images of basal foot shown in (**H**, dotted box). The outer limiting membrane (**J,K**, black arrowhead) and the basal feet of Müller glia **(L,M)** are strongly stained. Amacrine and ganglion cells show faint TNFα-positive signal **(H,I)**. n: nuclei of cone photoreceptors (ns, *p* > 0.05; ^∗^*p* < 0.05).

Proliferation of Müller glia and rod precursors was maximal at 5 wpf (Figure 2I), suggesting that dying photoreceptors secrete a signal to induce proliferation. Several molecules including TNFα are known to stimulate re-entry of Müller glia into the cell cycle in zebrafish (Wan et al., 2012, 2014; Lenkowski et al., 2013; Nelson et al., 2013; Zhao et al., 2014; Gorsuch et al., 2017). To evaluate TNFα expression in *gosh* mutants, we performed qPCR at 3, 5 and 7 wpf (Figure 4A). 3 wpf of control and *gosh* samples showed baselines levels of TNFα. While, 5 and 7 wpf *gosh* samples displayed an increased in TNFα mRNA relative to control samples. Next, we evaluated immunolocalization of TNFα in *gosh* mutant retinas at 3 and 5 wpf, since proliferation of Müller glia does not occur at 3 wpf, but was highly activated at 5 wpf. At 3 wpf, TNFα expression was not detected in either wild-type or *gosh* mutant retinas (Figures 4B,C). At 5 wpf, TNFα was still undetectable in wild-type retinas (Figure 4D); however, *gosh* mutant photoreceptors exhibited TNFα expression (Figure 4E, arrows). Since TNFα also promotes amplification of proliferating Müller glia during regeneration (Nelson et al., 2013), we evaluated TNFα expression at 7 wpf. In wild type, TNFα expression was not observed (Figures 4F,G). However, in *gosh* mutants, TNFα expression was detected in dying cone photoreceptors (Figures 4H–K). In addition, Müller cells also expressed TNFα at 7 wpf in *gosh* mutants (Figures 4H,I,L,M). These data resemble those of light-induced retinal damage, in which TNFα is initially expressed in dying photoreceptors and subsequently in Müller glia (Nelson et al., 2013).

### Proliferating Müller Glia Express Sox2 and NPCs Express Pax6 in *gosh* Mutant Retinas

**FIGURE 5:**
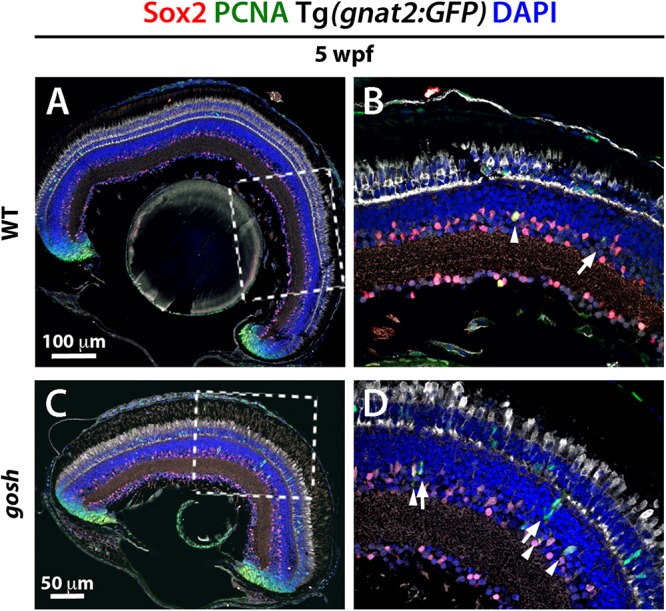
Müller glia express Sox2 during the regeneration response in *gosh* mutants. Wild-type and *gosh* mutant retinas at 5 wpf labeled with anti-Sox2 and anti-PCNA antibodies, and DAPI. Tg(*gnat2:GFP*) transgene visualizes cones. Wild-type retinas display Sox2 expression in the CMZ and amacrine cells (**A,C**, dotted boxes show the area magnifies in **B,D**). Müller glia exhibit faint expression of Sox2. At 5 wpf, *gosh* mutant retinas show that Sox2 expression is colocalized with PCNA in the INL (**D**, arrowheads) probably in Müller glia, whereas subsequently differentiating NPCs express only PCNA (**D**, arrows).

Several transcription factors are essential to reprogram Müller glia to become stem cell-like in damaged retina. Sox2 and Pax6 are well-known retinal progenitor markers (Raymond et al., 2006; Thummel et al., 2010; Gorsuch et al., 2017). Since we detected a regenerative response in *gosh* mutants at 5 wpf, we evaluated Sox2 and Pax6 expression at this developmental stage. Five-wpf wild-type sibling retinas displayed strong Sox2 immunoreactivity in the CMZ, amacrine cells in the INL, displaced amacrine cells in the ganglion cell layer (GCL), and weak expression of Sox2 in Müller glia (Figures 5A,B). *gosh* mutant retinas showed increased Sox2 expression in PCNA-positive Müller glia, with fusiform shaped nuclei in the INL (Figures 5C,D). It is noteworthy that NPCs were Sox2-negative.

**FIGURE 6:**
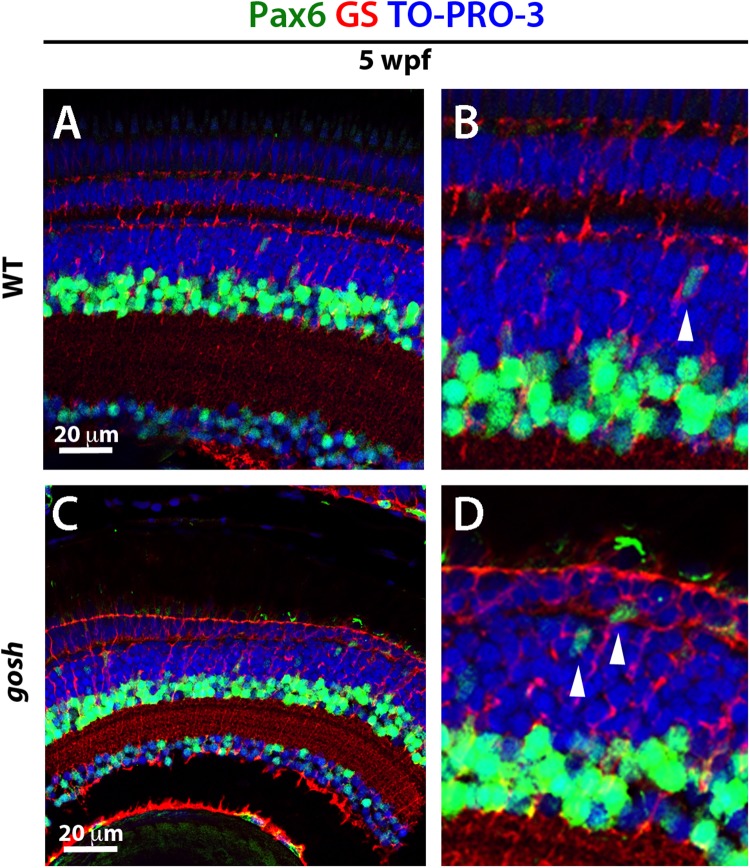
NPCs express Pax6 during the regenerative response in *gosh* mutants. Wild-type and *gosh* mutant retinas labeled with antibodies against Pax6 and GS, which are expressed in NPCs and Müller glia, respectively. In wild-type retinas, Pax6 is observed in amacrine cells and retinal ganglion cells **(A,B)**. Weaker signals were observed in NPCs migrating to the ONL (**B**, arrowhead). Five-wpf *gosh* mutant retinas show that Pax6 is expressed in amacrine cells and retinal ganglion cells, as well as probable NPCs migrating toward the ONL (**C,D**, arrowheads).

We also examined Pax6 expression in wild-type sibling and *gosh* mutant retinas during the regenerative response at 5 wpf. In wild-type retinas, Pax6 expression was observed in amacrine and ganglion cells (Figures 6A,B), with reduced expression in dividing NPCs migrating to the ONL (Figures 6B, arrowhead). It is noteworthy that Pax6 is not expressed in GS-positive Müller glia. *gosh* mutant retinas exhibited Pax6 expression in amacrine and ganglion cells, as well as in some cells migrating toward the ONL. Based on their location, these are likely to have been NPCs (Figures 6C,D). Again, we did not observe coexpression of GS and Pax6 in *gosh* mutant retinas. Thus, in *gosh* mutant retinas, proliferating Müller glia express Sox2 and Müller glia-derived NPCs express Pax6.

### TNFα Promotes Proliferation of Müller Glia at 3 wpf in *gosh* Mutants

**FIGURE 7:**
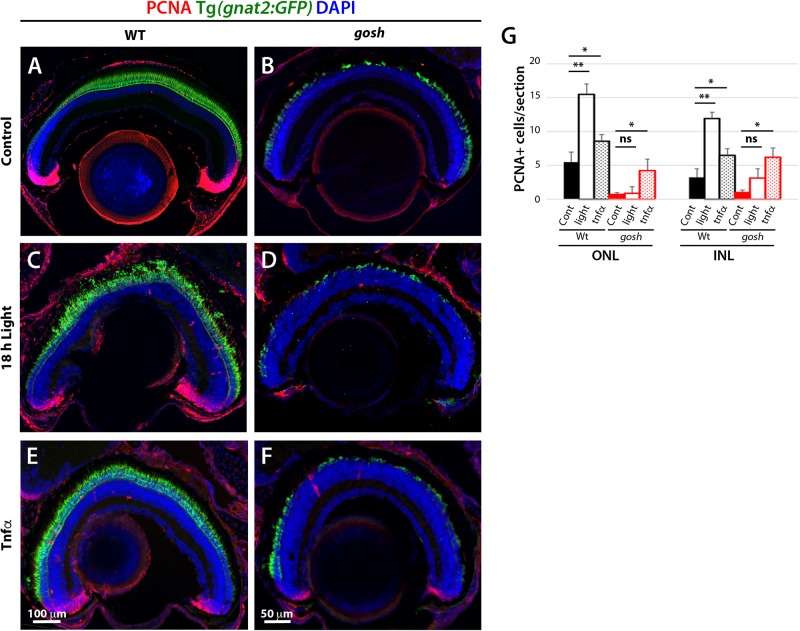
Proliferation of Müller glia and rod precursor cells is not activated by light-induced damage, but by TNFα in *gosh* mutants at 3 wpf. Wild-type and *gosh* mutant retinas at 3 wpf are labeled with anti-PCNA antibody. Nuclei are stained with DAPI. The transgene Tg(*gnat2:GFP*) visualizes cone photoreceptors. In wild-type, undamaged controls, PCNA is expressed in the CMZ, and PCNA-positive cells are observed in the INL and ONL **(A)**. However, the PCNA signal is almost absent in *gosh* mutant retinas **(B)**. Treatment for 18 h under intense light damages photoreceptors **(C,D)**. Wild-type sibling retinas showed an increase in the number of PCNA-positive cells located in the INL and ONL. On the other hand, the PCNA signal is still absent in *gosh* mutant retinas. TNFα intravitreal injections increase the number of PCNA-positive cells in wild-type sibling and *gosh* mutant retinas **(E,F)**. Histogram depicting the number of PCNA-positive cells **(G)**. Bars and lines indicate mean ± SEM, n: 6*–*11. Black and red bars, wild-type siblings and *gosh* mutants. Filled bars, no treatment; Open bars, photodamaged samples; Dotted bars, TNFα-injected sample (ns, *p* > 0.05; ^∗^*p* < 0.05; ^∗∗^*p* < 0.01).

Since Müller glia exhibit a gliotic response without proliferation by 3 wpf (Figures 2, 3), we evaluated whether Müller glia could be shifted to a proliferative response by additional acute damage. We introduced photoreceptor damage using high-intensity light exposure in 3 wpf zebrafish. In damaged wild-type sibling retinas, photoreceptor integrity was compromised, resulting in disorganization of the photoreceptor layer (Figures 7A,C). PCNA signals were also elevated in these light-damaged wild-type retinas relative to undamaged control retinas (Figure 7G, Control ONL: 5.50 ± 1.50 and INL: 3.25 ± 1.25; light damaged ONL: 15.53 ± 1.54, and INL: 11.17 ± 0.97), suggesting that this light treatment induced a proliferative response. In *gosh* mutants, light-induced damage increased degeneration of the photoreceptor layer compared to undamaged *gosh* mutants (Figures 7B,D). However, light-treated *gosh* retinas did not display a significant increase in the number of PCNA-positive cells relative to undamaged control *gosh* mutant retinas (Figure 7G, control *gosh* ONL: 0.71 ± 0.31, and INL: 1.00 ± 0.38; light-treated *gosh* ONL: 0.91 ± 0.98, and INL: 3.13 ± 1.40). These results suggest that while this light-mediated damage successfully compromised structural integrity of photoreceptors in both wild-type and *gosh* mutant retinas at 3 wpf, it failed to induce a regenerative response in *gosh* mutant retinas.

TNFα is expressed in dying cones when a proliferation response occurred in *gosh* mutants at 5 wpf (Figure 4). Accordingly, we examined whether introduction of TNFα into the eye induces a proliferative response in *gosh* mutants at 3 wpf. Two rounds of intravitreal injections of TNFα were performed at 3 wpf in wild-type and *gosh* mutant fish every 12 h. TNFα treatment of wild-type sibling retinas caused a significant increase in the number of PCNA-positive cells in ONL and INL (Figures 7E,G, untreated ONL: 5.50 ± 1.50, and INL: 3.25 ± 1.25; TNFα treated ONL: 8.60 ± 0.85, and INL: 6.50 ± 0.87). Similarly, TNFα treatment significantly increased the number of PCNA-positive cells in the ONL and INL of *gosh* mutants (Figures 7F,G, untreated ONL: 0.71 ± 0.31, and INL: 1.00 ± 0.38; TNFα treated ONL: 4.25 ± 1.70, and INL: 6.25 ± 1.38). These results suggest that at early stages (3 wpf), Müller glia of *gosh* mutants exist in a primed proliferative state that requires an additional signal, such as TNFα, to enter a proliferative state.

## Discussion

This study demonstrated that in chronic photoreceptor degeneration mutants, *gosh* exhibited a peak of photoreceptor apoptosis at 2–3 wpf, without inducing proliferation of Müller glia and rod precursors. Furthermore, we observed that Müller glia and rod precursors undergo cell-cycle progression in *gosh* mutants after 5 wpf. This proliferative response was correlated with expression of TNFα in dying photoreceptors, Sox2 expression in proliferating Müller glia, and Pax6 expression in NPCs, as previously described for acute light-induced photoreceptor damage. Finally, ectopic introduction of TNFα significantly increased the proliferative response in *gosh* mutant retinas at 3 wpf. Thus, our findings clearly indicate that there is a transition of Müller glia from non-proliferative gliosis to a regenerative state in the presence of gliosis in zebrafish chronic photoreceptor degeneration *gosh* mutants. TNFα can bypass this transition and promotes Müller cells to exit non-proliferative gliosis and to initiate a regenerative response, even at early stages of photoreceptor degeneration.

Degenerating cone or rod photoreceptors are able to regenerate in zebrafish at 7 dpf (Morris et al., 2008). However, *gosh* mutants do not show retinal regeneration until 5 wpf. It is important to understand why this time-lag between photoreceptor degeneration and regeneration exists in *gosh* mutants. In *pde6c^*w*59^* mutants, severe cone cell death is induced, which promptly activates a regenerative response of Müller glia at 7 dpf. In light-induced acute damage, which eliminates both rod and cone photoreceptors, a proliferative response of Müller glia occurs promptly (Kassen et al., 2007). In the transgenic line Tg(*Xops:mCFP*), rod cell death also occurs in an acute process. In this case, only rod precursor cells proliferate. Interestingly, using cell ablation with rod-specific expression of nitroreductase (NTR), the number of proliferating Müller glia was variable, depending on the extent of rod photoreceptor loss (Montgomery et al., 2010). For example, ablating only a subset of rods in Tg(*zop:nfsB-EGFP*)^nt20^ retinas stimulates only rod precursor proliferation, but not Müller glia proliferation, while loss of all rods in Tg(*zop:nfsB-EGFP*)^nt19^ retinas induces robust Müller glial proliferation. Thus, depending on the cell type or the number of damaged photoreceptors, Müller cells or rod precursor cells activate different regenerative responses. Since *gosh* mutants show slow degeneration of cones and rods at 1–3 wpf, it is possible that such slow photoreceptor damage does not reach the threshold necessary to induce a proliferative response.

How cell types and the extent of retinal damage are monitored to activate appropriate retinal regeneration mediated by Müller cells or rod precursor cells? Recently, it was reported that regulatory T-cells are recruited into damaged tissues including spinal cord, retina, and heart in zebrafish, and stimulate tissue regeneration through secretion of organ-specific regenerative factors, for example Igf1 in the retina (Hui et al., 2017). It was also reported that microglia-mediated inflammation is required for neuronal regeneration in response to trauma brain injury in zebrafish (Kyritsis et al., 2012), and that microglia control Müller cell responsiveness to photoreceptor loss (White et al., 2017). These findings suggest that immune cells, such as microglia and regulatory T cells, are important for initiating regeneration by Müller glia in the retina. Clearance of dead or dying neurons by microglia and secretion of growth factors for Müller cells by regulatory T cells may be important to transform Müller glia from non-proliferative gliosis to a proliferative/regenerative state. As another possible mechanism, there are several factors that control the degree of activation of Müller glia. In response to acute light damage of the retina, only ∼50% of Müller cells dedifferentiate and proliferate, while the rest remain as differentiated glia. Notch (Conner et al., 2014), Let-7 (Ramachandran et al., 2010), Dkk (Ramachandran et al., 2011), TGFβ (Lenkowski et al., 2013), and Insm1a (Ramachandran et al., 2012) are involved in induction and maintenance of this quiescent Müller glia population. *gosh* mutant retinas may express such inhibitory molecules at 2–3 wpf, which prevent Müller glia reprograming and reentry into the cell cycle. Further studies will be necessary to determine the underlying mechanism.

In *gosh* mutants, Müller glia do not start cell-cycle progression at 3 wpf, but show a non-proliferative gliotic response, which occurs mostly in mammalian nervous system damage. It is important to understand the different mechanisms by which non-proliferative gliosis and proliferative gliosis/regenerative responses are activated in Müller glia after photoreceptor degeneration. In mammals, after retinal damage, Müller cells exhibit a reactive gliotic response, featuring cell hypertrophy and upregulation of GFAP (Iribarne et al., 2008; Bringmann and Wiedemann, 2012). This reactive gliosis is initially neuroprotective, but eventually leads to loss of retinal neurons and causes scarring. In zebrafish, Müller cells transiently display reactive gliosis even in response to light-induced retinal damage (Thomas et al., 2016), suggesting that this gliotic response generally occurs prior to Müller cell proliferation, regardless of the extent of damage. Ectopic introduction of TNFα can induce proliferation of Müller glia at 3 wpf in *gosh* mutants, suggesting that Müller glia already have the potential to exit non-proliferative gliosis and to initiate a proliferative/regenerative response at early stages of photoreceptor degeneration.

During light-induced retinal damage, TNFα expression is initially increased in apoptotic photoreceptors and later in Müller glia. Knockdown of TNFα significantly reduces proliferation of Müller glia in light-induced retinal damage (Nelson et al., 2013). These observations suggest that TNFα induces Müller glia to re-enter the cell cycle. Indeed, we found that ectopic introduction of TNFα increases the number of proliferating Müller glia in *gosh* mutants even at 3 wpf, when primarily non-proliferative gliosis occurs. However, the number of proliferating Müller cells is still lower than in *gosh* mutants at 5 wpf. Thus, it is likely that additional factors function synergistically with TNFα to promote Müller glia proliferation or to suppress inhibitors of Müller glial proliferation, such as Notch3 or Dkk (Ramachandran et al., 2011; Conner et al., 2014). As discussed before, microglia are reactive during retinal damage (Mitchell et al., 2018); however, microglia do not express TNFα during light-induced retinal damage (Nelson et al., 2013). It is necessary to determine whether microglia are required for TNFα activation. Another interesting point is that *gosh* mutants activate regenerative proliferation at 3 wpf in response to TNFα treatment, but not to intense photostimulation, although the illumination is sufficient to trigger regenerative proliferation in wild-type controls (Figure 7G). It remains unclear why *gosh* mutants cannot regenerate at 3 wpf, even with this additional damage. One explanation could be that the magnitude of the damage was not enough to reach the threshold. Perhaps, introducing longer or more serious damage could eventually overcome the halt and Müller glia could enter the cell cycle. Additionally, since a large fraction of photoreceptors are already eliminated in *gosh* mutants by 3 wpf (Figure 1E) and TNFα expression is not induced in 3 wpf *gosh* mutants (Figure 4C), it might be that the additional damage by intense illumination to the remaining photoreceptors does not reach the threshold to initiate regenerative proliferation. On the other hand, application of TNFα seems to exceed the threshold for regenerative proliferation in *gosh* mutants, suggesting a role of TNFα in regenerative proliferation.

Most of our understanding of molecules that induce retinal regeneration in zebrafish was developed using acute damage models. Furthermore, acute damage has often been applied to adult zebrafish. Few studies using zebrafish embryos have employed genetic mutations (Morris et al., 2008; Meyers et al., 2012). During retinal regeneration, many genes of Müller glia exhibit altered expression profiles in connection with cell cycle re-entry (Kassen et al., 2007; Craig et al., 2008; Qin et al., 2009; Ramachandran et al., 2012). Among these, reprograming factors such as Ascl1a, Lin28a, and Stat3 were identified. In addition, Sox2 regulates the early reprograming process of Müller glia in light-induced damage (Gorsuch et al., 2017). Sox2 is also required for expression of Ascl1a and Lin28a, but not of Stat3. Pax6 is not required for Müller cell proliferation, but is for subsequent NPC proliferation (Thummel et al., 2010). Sox2 and Pax6 were detected in Müller glia and Müller glia-derived NPCs in *gosh* mutants at 5 wpf, suggesting that at least these factors are involved in this chronic injury model. Further studies are required to reveal molecular mechanisms underlying regeneration associated with chronic retinal damage. *gosh* mutants provide a useful model for studying retinal regeneration in chronic photoreceptor degeneration, and for developing regenerative therapies to treat human patients suffering from photoreceptor degeneration.

## Data Availability Statement

All datasets generated for this study are included in the article/Supplementary Material.

## Ethics Statement

All zebrafish experiments performed at the Okinawa Institute of Science and Technology Graduate School (OIST) were carried out in accordance with the OIST Animal Care and Use Program, which is based on the Guide for the Care and Use of Laboratory Animals by the National Research Council of the National Academies and which is accredited by the Association for Assessment and Accreditation of Laboratory Animal Care (AAALAC International). All experimental protocols were approved by the OIST Institutional Animal Care and Use Committee (Approval ID: 2014-83∼86). All experiments performed at the University of Notre Dame were approved by the animal use committee at the University of Notre Dame and comply with the ARVO statement for the use of animals in vision research.

## Author Contributions

MI and IM conceived this study. MI carried out the experiments, analyzed the data, and prepared the manuscript. DH and IM edited the manuscript.

## Conflict of Interest

The authors declare that the research was conducted in the absence of any commercial or financial relationships that could be construed as a potential conflict of interest.
