# Supplementary material for: TNFα Induces Müller Glia to Transition From Non-proliferative Gliosis to a Regenerative Response in Mutant Zebrafish Presenting Chronic Photoreceptor Degeneration
Source: Front Cell Dev Biol. 2019 Nov 26;7:296. doi: 10.3389/fcell.2019.00296 (PMC6962764; doi:10.3389/fcell.2019.00296)
Supplement: Supplementary file 1 [file Data_Sheet_1.zip › Supplementary Figures Iribarne.docx]

**Supplementary Materials**

**TNFα induces Müller glia to transition from non-proliferative gliosis to a regenerative response in mutant zebrafish presenting chronic photoreceptor degeneration**

Maria Iribarne, David R. Hyde, and Ichiro Masai

**Supplementary Figures: Figure S1-4**

##
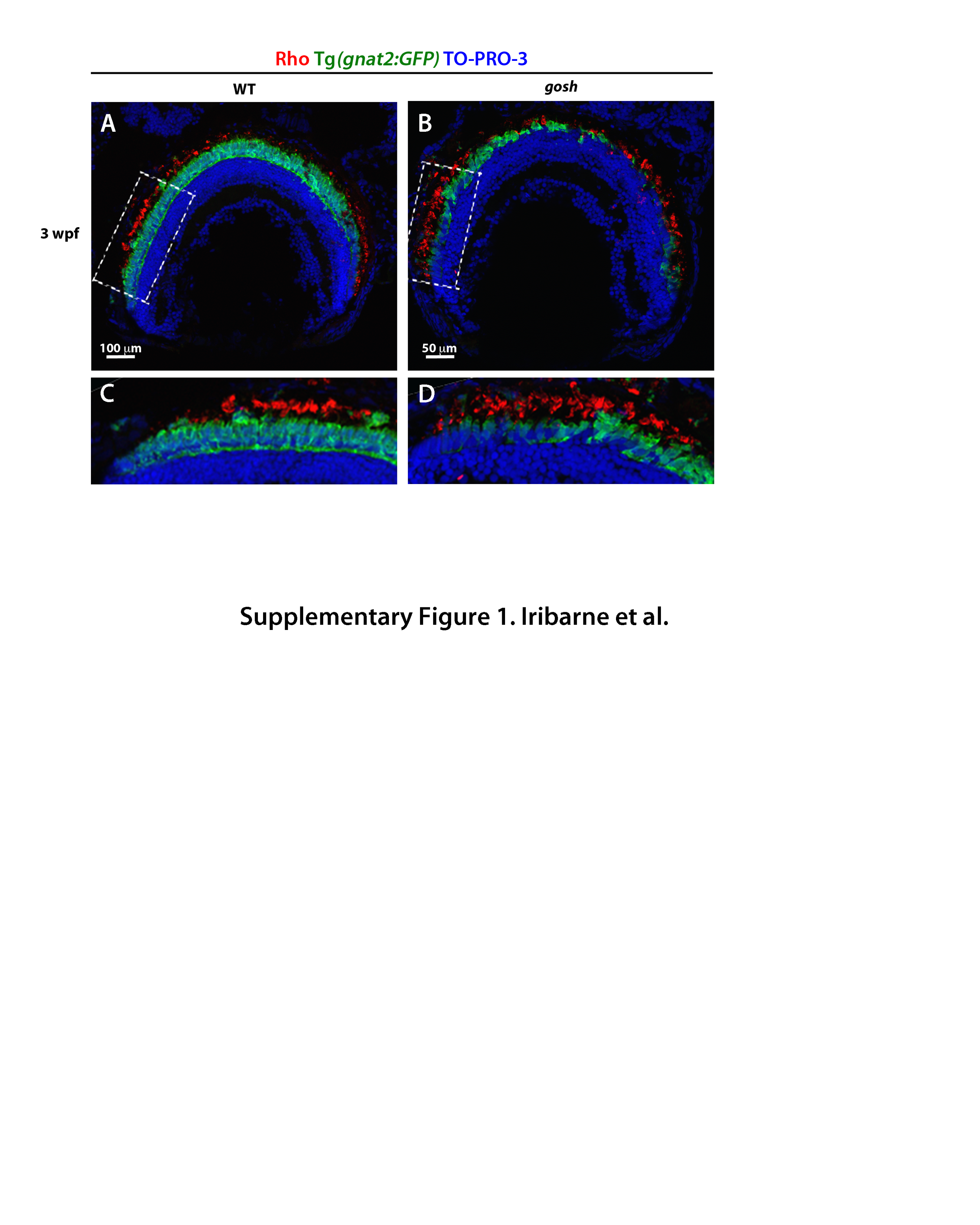


## Supplementary Figure 1. *gosh* mutants show photoreceptor degeneration at 3 wpf.

Labeling of wild-type and *gosh* mutant retinas at 3 wpf with rhodopsin antibody (red). The transgenic line Tg*(gnat2:GFP)* was used to monitor cone photoreceptors. Nuclei were labeled with TO-PRO-3. In wild-type central retinas, the ONL thickens to segregate rod and cone nuclear layers (A, C). Cones are highly elongated. Rhodopsin is localized in the OS. In *gosh* mutant central retinas, the ONL is very thin, and cones are both fewer in number and abnormal in shape (B, D). Rhodopsin is hardly observed in the central retina. The CMZ, where progenitors proliferate to form all types of retinal neurons, shows nascent cones (C). The *gosh* CMZ presents relatively normal cone photoreceptors (D). Dotted boxes in A and B show the area magnified in C and D, respectively.

##
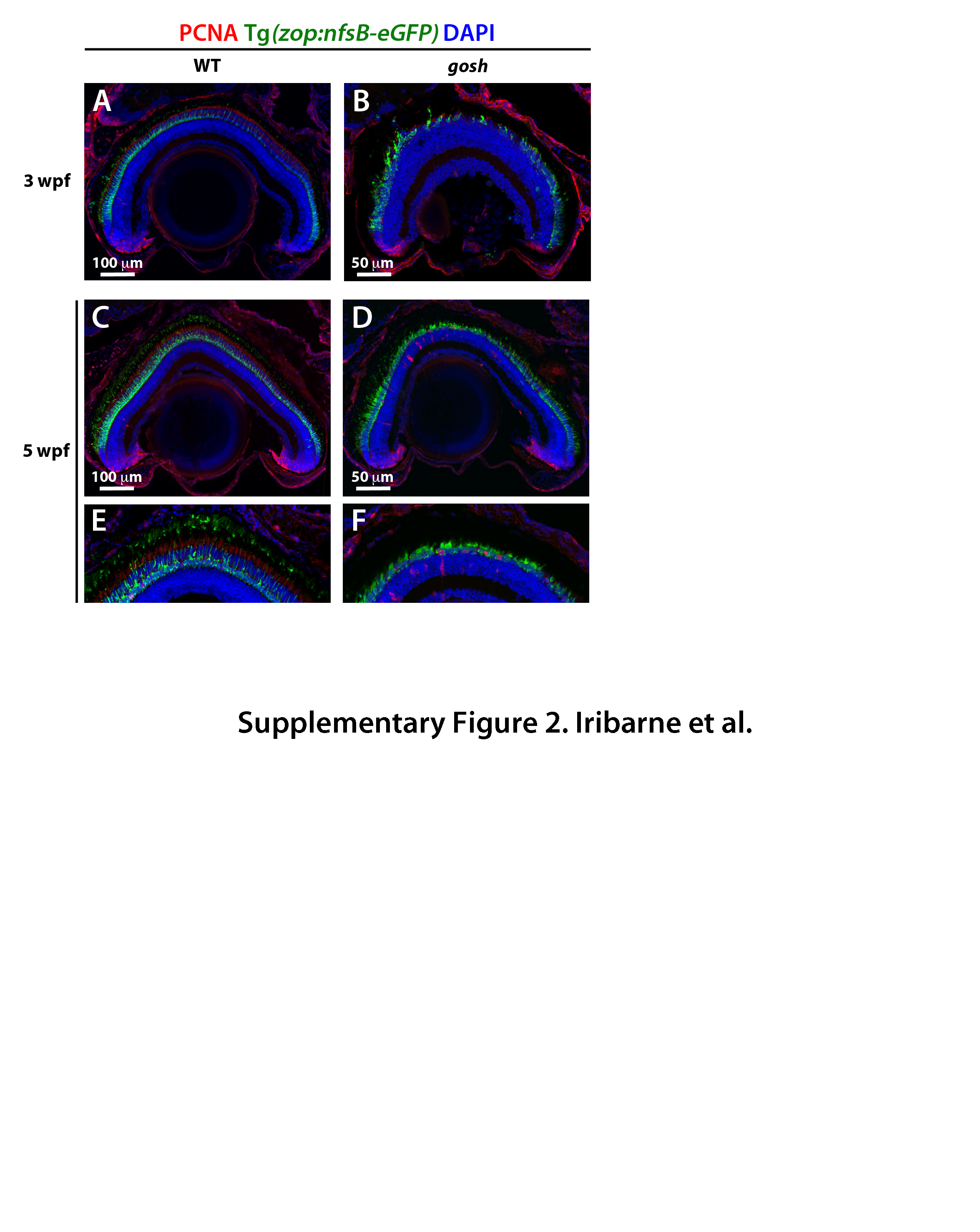


## Supplementary Figure 2. Rod photoreceptors degenerate in *gosh* mutants at 3 wpf.

3- and 5-wpf wild-type and *gosh* mutant retinas combined with the transgene Tg(*zop:nfsB-eGFP*), which visualizes rod photoreceptors. Proliferation is monitored with PCNA immunostaining (red), and nuclei are counter-stained with DAPI (blue). In 3-wpf, wild-type retinas show GFP expression in rod photoreceptors in the ONL (A). *gosh* mutants displayed abnormal rod cell shape and a reduced number of rods (B). By 5 wpf, *gosh* rod photoreceptors start to recover, as evident from the increase in PCNA labeling and rod cell shape (D, F).


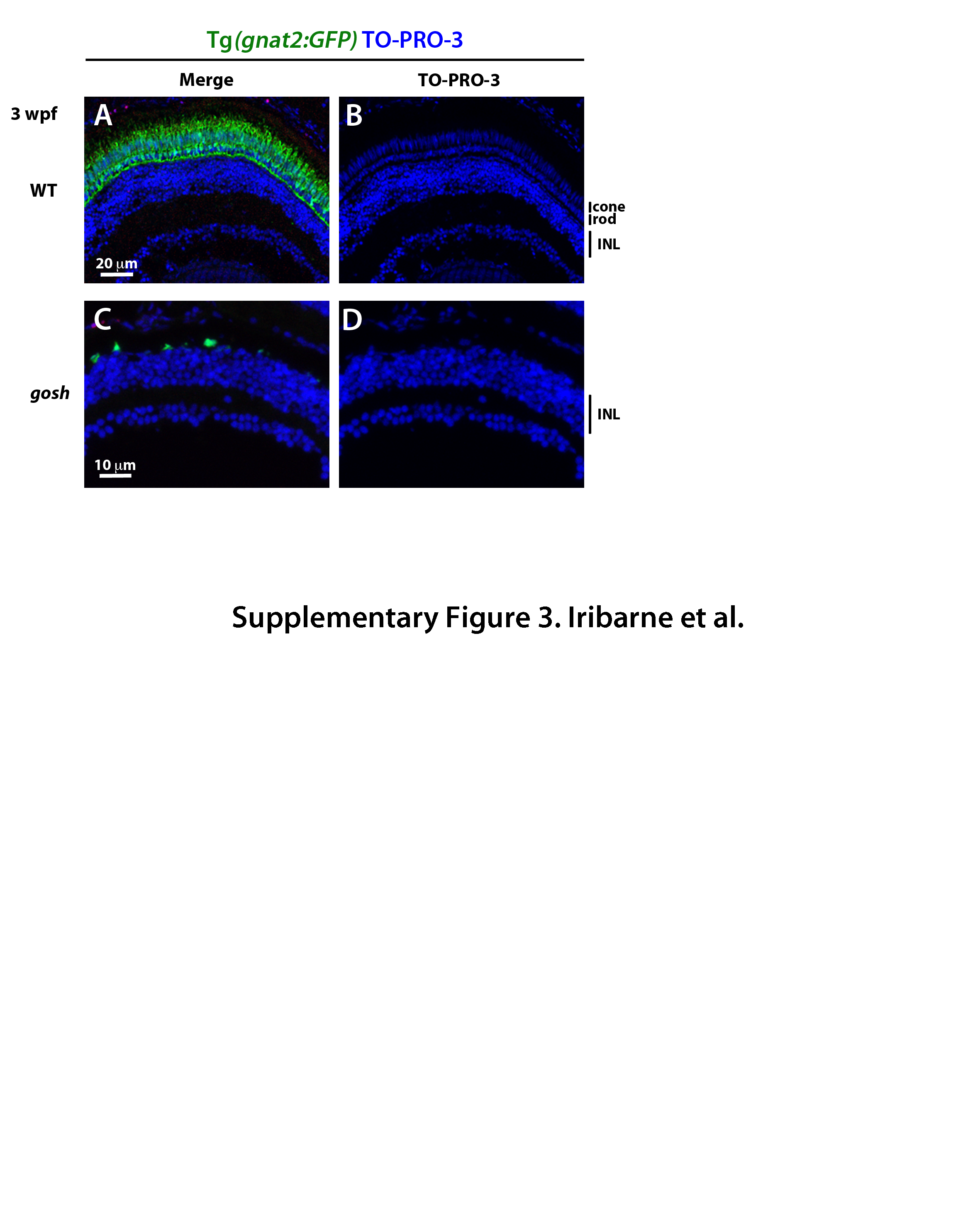


## Supplementary Figure 3. In *gosh* mutants, cone photoreceptors degenerate at 3 wpf.

3-wpf central retinas of wild type and *gosh* mutants combined with the transgene Tg*(gnat2:GFP*), which visualizes cone photoreceptors. Nuclei are counter-stained with TO-PRO-3 (blue). In wild-type retinas, GFP expression is observed in cone photoreceptors. *gosh* mutants displayed fewer cones with abnormal cone cell shapes. The TO-PRO-3 channel shows that rods and cones are segregated in wild-type retinas, but that in *gosh* mutants, cone and rod photoreceptors fail to form distinct layers.


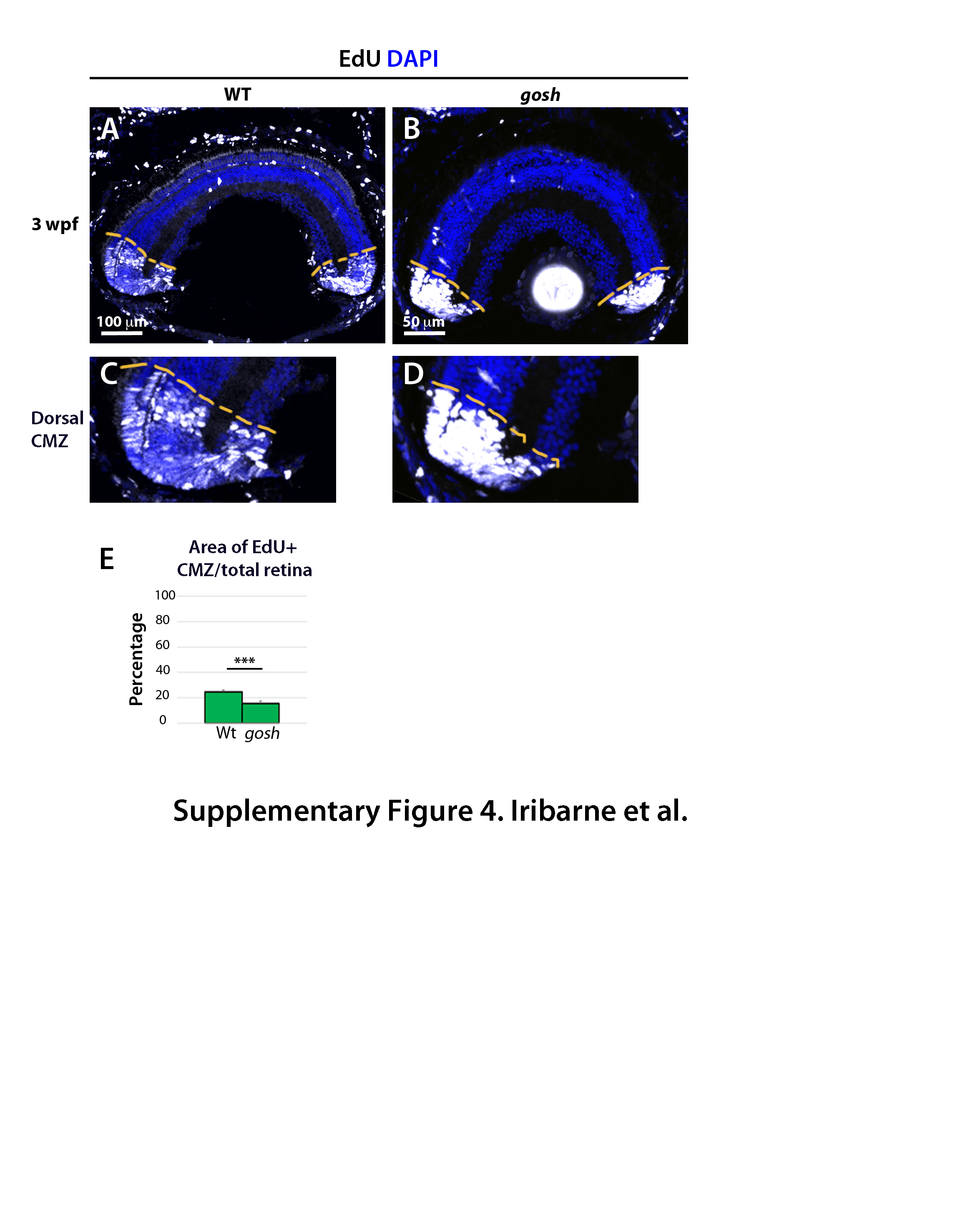


## Supplementary Figure 4. Wild type and *gosh* mutants incorporate EdU into nascent cells from the CMZ at 3 wpf.

3-wpf retinas of wild type (A, C) and *gosh* mutants (B, D) pulse chase after 3 days to display new cells. Nuclei are counter-stained with DAPI (blue). Both wild type and *gosh* retinas incorporated the nucleoside analog, EdU into new cells in CMZ-derived cells. However, *gosh* mutant retinas present a reduced area of EdU-positive staining over the total size of the retina compare to wild-type retinas (E). Notably, cells derived from Müller glia are present in wild-type retinas, while fewer EdU-positive cells are observed in *gosh* retinas.
